# Predictors of mortality of Hypertensive Intracerebral Hemorrhage (HICH) patients: a single centre study

**DOI:** 10.1186/s12883-026-04847-z

**Published:** 2026-04-09

**Authors:** Lisda Amalia, Shafa Triani Fadhillah, Iin Pusparini, Achmad Adam, Marda Arif Furqani

**Affiliations:** 1 Department of Neurology, Faculty of Medicine, Universitas Padjadjaran – Dr. Hasan Sadikin General Hospital, Bandung, Indonesia; 2 Faculty of Medicine, Universitas Padjadjaran, Bandung, Indonesia; 3 Department of Neurosurgery, Faculty of Medicine, Universitas Padjadjaran – Dr. Hasan Sadikin General Hospital, Bandung, Indonesia; 4 Jl.Eykman 38, Bandung, 40161 Indonesia

**Keywords:** Hypertensive Intracerebral Hemorrhage (HICH), Mortality, Diabetes mellitus, NIHSS, Bleeding location, ICH Score

## Abstract

**Introduction:**

Hypertensive Intracerebral Hemorrhage (HICH) is one of the leading causes of death and disability in the world, with a high mortality rate. Various demographic and clinical factors influence mortality in ICH patients.

**Objective:**

This study aims to identify the factors that affect in-hospital mortality in patients with hypertensive intracerebral hemorrhage (HICH).

**Methods:**

This study used a retrospective cohort design with medical record data from hypertensive intracerebral hemorrhage (HICH) patients at the Neurology Department of Dr. Hasan Sadikin Hospital, Bandung between January 2023 until December 2024. Univariate analysis was performed to describe sample characteristics, bivariate analysis with chi-square test was used to test the relationship between independent variables and mortality, and multivariate analysis with logistic regression was applied to identify the factors that most influenced mortality.

**Results:**

Among the 177 patients analyzed, 64 (36.16%) died. Factors that were significantly associated with mortality were diabetes mellitus (*p* = 0.005 OR = 5.481), NIHSS score (*p* = 0.003 OR = 3.589), bleeding location (*p* < 0.001 OR = 10.494), and ICH score (*p* = 0.002 OR = 2.757).

**Conclusion:**

Diabetes mellitus, NIHSS score, infratentorial location of bleeding, and ICH score are the primary factors contributing to mortality in hypertensive intracerebral hemorrhage (HICH) patients. Early identification and optimal management of these factors can help improve patient clinical outcomes.

**Supplementary Information:**

The online version contains supplementary material available at 10.1186/s12883-026-04847-z.

## Introduction

Hypertensive intracerebral hemorrhage (HICH) is a serious and life-threatening medical condition with a high mortality rate. HICH represents one of the most severe complications of hypertension [1]. Hypertensive intracerebral hemorrhage accounts for approximately 50%–70% of all cases of spontaneous intracranial hemorrhage [2]. The mortality rate of HICH ranges from 34% to 43.9% and increases to 47% after one year. Hypertensive intracerebral hemorrhage (HICH) results from chronic high blood pressure causing lipohyalinosis, fibrinoid necrosis, and Charcot-Bouchard microaneurysms in small, deep penetrating arteries. Rupture of these vessels causes hematoma formation in the basal ganglia, thalamus, or pons, leading to mass effect, increased intracranial pressure (ICP), and secondary perihematomal edema [2, 10]. Effective management of patients with HICH, particularly in addressing poor clinical outcomes, requires a comprehensive understanding of the various risk factors involved. Early identification of mortality risk factors in HICH is crucial to improving patient outcomes [3]. 

Both demographic and clinical factors influence the mortality of patients with hypertensive intracerebral hemorrhage (HICH). Demographic factors include age and sex [4, 5]. Clinical factors consist of several variables significantly associated with HICH mortality, including hypertension [6], diabetes mellitus, hyperglycemia [7], hematoma volume, hematoma location [8], National Institutes of Health Stroke Scale (NIHSS) [9], and ICH score [10]. Levels of low-density lipoprotein (LDL), hemoglobin (Hb), and several coagulation parameters such as prothrombin time (PT), activated partial thromboplastin time (APTT), and international normalized ratio (INR) have also been reported to be associated with HICH mortality in previous studies [11–13]. Therapeutic interventions, including the administration of antihypertensive drugs, glucose management, antilipidemic agents, and surgical procedures, have been associated with HICH mortality [14, 15]. 

Factors influencing mortality in patients with hypertensive intracerebral hemorrhage (HICH) vary across different regions and populations. Previous studies have reported inconsistent findings regarding the most significant predictors of mortality. However, specific research investigating these factors at Dr. Hasan Sadikin General Hospital is currently lacking. Moreover, it remains unclear whether risk factors identified in international studies are equally relevant to the Indonesian population. Therefore, this study aims to analyze factors associated with mortality among HICH patients at Dr. Hasan Sadikin General Hospital.

## Methods

This study employed a retrospective cohort design with an observational analytic approach. The data were obtained from secondary sources, specifically medical records of patients with hypertensive intracerebral hemorrhage (HICH) treated at Dr. Hasan Sadikin General Hospital, Bandung between January 2023 until December 2024. A total sampling technique was used, resulting in 177 eligible medical records. Inclusion criteria were as follows: (a) confirmed diagnosis of HICH by computed tomography (CT) scan, (b) age > 18 years, (c) symptom onset within ≤ 24 h prior to admission, and (d) complete documentation, including laboratory findings, therapeutic interventions, and/or surgical procedures, and (e) bleeding location (supratentorial or infratentorial). Exclusion criteria included: (a) HICH due to trauma identified through patient history, (b) underlying brain tumors or vascular malformations confirmed by MRI, MRA, or CTA, (c) known hematologic disorders based on laboratory results, (d) non-neurological complications (e.g., pulmonary infections, urinary tract infections, electrolyte imbalances, or deep vein thrombosis), and (e) pregnancy at the time of HICH diagnosis. Data analysis consisted of univariate analysis to describe sample characteristics, bivariate analysis using the Chi-square test to assess the relationship between independent variables and mortality, and if the bivariate analysis is statistically significant (p, 0.05), then it will be continued with multiple logistic regression analysis to see the factors that most influence mortality in patients with intracerebral hemorrhage. Statistical significance was set at *p* < 0.05.

## Results

Based on data obtained from the Department of Neurology, Dr. Hasan Sadikin General Hospital, a total of 177 cases of hypertensive intracerebral hemorrhage (ICH) were analyzed (100%). Among these, 64 patients (36.16%) died, while 113 (63.84%) survived (Table 1).

**Table 1:** Demographic characteristic of research subjects

Demographic Characteristics	“Alive” (*n*,%)	“Deceased” (*n*,%)	*p*-value
Age			
18–24	0 (0)	0 (0)	0.924
25–44	12 (66,7)	6 (33,3)	
45–60	55 (62,5)	33 (37,5)	
61–90	46 (64,8)	25 (35,2)	
Sex			
Male	49 (27,7)	33 (18,6)	0.371
Female	64 (36,2)	31 (15,5)	

Based on the bivariate analysis presented in Table 2, several clinical factors showed a potentially significant association with in-hospital mortality and were therefore considered for multivariate analysis. These factors include diabetes mellitus (*p* < 0.001), fasting blood glucose (FBG) (*p* = 0.007), and hemoglobin (HB) level (*p* < 0.001). Additionally, coagulation parameters such as prothrombin time (PT) (*p* < 0.001), international normalized ratio (INR) (*p* = 0.016), and activated partial thromboplastin time (APTT) (*p* = 0.047) were also associated with mortality risk. Other significant factors were National Institutes of Health Stroke Scale (NIHSS) score (*p* < 0.001), bleeding volume (*p* = 0.038), bleeding location (*p* < 0.001), ICH score (*p* < 0.001), use of antihypertensive drugs (*p* < 0.001), and use of antilipidemic agents (*p* < 0.001). Conversely, variables such as systolic blood pressure (SBP) (*p* = 0.598), diastolic blood pressure (DBP) (*p* = 0.633), mean arterial pressure (MAP) (*p* = 0.112), low-density lipoprotein (LDL) level (*p* = 0.124), white blood cell (WBC) count (*p* = 0.625), platelet count (*p* = 0.362), use of glucose-lowering drugs (*p* = 0.800), and surgical intervention (*p* = 0.067) showed no significant association with mortality in this study. Therefore they were excluded from the multivariate analysis.

**Table 2:** Clinical characteristics of patients with Hypertensive Intracerebral Hemorrhage (HICH)

Clinical Characteristics	“Alive” (*n*,%)	“Deceased” (*n*,%)	*p*-value
SBP			
Normal	0 (0)	1 (100)	0.598
Prehypertension	6 (60)	4 (40)	
Hypertension Stage 1	21 (65,6)	11 (34,4)	
Hypertension Stage 2	86 (64,2)	48 (35,8)	
DBP			
Normal	3 (42,9)	4 (57,1)	0.633
Prehypertension	13 (68,4)	6 (31,6)	
Hypertension Stage 1	22 (61,1)	14 (38,9)	
Hypertension Stage 2	75 (65,2)	40 (35,8)	
MAP			
Normal	0 (0)	1 (100)	0.112
Prehypertension	4 (100)	0 (0)	
Hypertension Stage 1	11 (50)	11 (50)	
Hypertension Stage 2	98 (65,3)	52 (34,7)	
Diabetes			
Yes	12 (35,3)	22 (64,7)	< 0.001
No	101 (70,6)	42 (29,4)	
Fasting blood glucose			
Low	1 (100)	0 (0)	0.007
Normal	89 (70,6)	37 (29,4)	
High	23 (46)	27 (54)	
LDL			
Optimal	50 (87,7)	7 (12,3)	0.124
Above Normal	43 (75,4)	14 (24,6)	
High Borderline	20 (57,1)	15 (42,9)	
High	13 (68,4)	6 (31,6)	
Very High	13 (68,4)	6 (31,6)	
WBC			
Normal	71 (62,3)	43 (37,7)	0.625
High	42 (66,7)	21 (33,3)	
Platelets			
Low	0 (0)	1 (100)	0.362
Normal	113 (62,4)	63 (35,8)	
Hemoglobin			
Normal	87 (73,7)	31 (26,3)	< 0.001
Low	26 (44,1)	33 (55,9)	
PT			
Low	1 (20)	4 (80)	< 0.001
Normal	95 (73,6)	34 (26,4)	
High	17 (39,5)	26 (60,5)	
INR			
Low	1 (100)	0 (0)	0.016
Normal	111 (65,7)	58 (34,3)	
High	1 (14,3)	6 (85,7)	
APTT			
Low	1 (25)	3 (75)	0.047
Normal	88 (68,8)	40 (31,3)	
High	24 (53,3)	21 (85,7)	
NIHSS			
No Stroke Symptoms	0 (0)	0 (0)	< 0.001
Minor Stroke	21 (87,5)	3 (12,5)	
Moderate Stroke	85 (78)	24 (22)	
Moderate to Severe Stroke	6 (16,2)	31 (83,8)	
Severe Stroke	1 (14,3)	6 (65,7)	
Bleeding Volume			
< 30 ml	99 (67,3)	48 (32,7)	0.038
≥ 30 ml	14 (46,7)	16 (53,3)	
Bleeding Location			
Infratentorial	7 (15,6)	38 (84,4)	< 0.001
Supratentorial	106 (80,3)	26 (19,7)	
ICH score			
0	58 (96,7)	2 (3,3)	< 0.001
1	36 (75)	12 (25)	
2	16 (34,8)	30 (65,2)	
3	3 (18.8)	13 (81,3)	
4	0 (0)	6 (100)	
5	0 (0)	0 (0)	
6	0 (0)	0 (0)	
Antihypertensive drugs			
Oral	69 (71,1)	28 (28,9)	< 0.001
IV	44 (55)	36 (45)	
Glucose lowering drugs			
Yes	11 (61,1)	7 (38,9)	0.800
No	102 (64,2)	57 (35,8)	
Antilipidemia agents			
Yes	48 (90,6)	5 (9,4)	< 0.001
No	65 (52,4)	59 (47,6)	
Surgery			
No surgery	108 (65,1)	58 (34,9)	0.067
Craniotomy	5 (62,5)	3 (37,5)	
VP shunt	0 (0)	3 (100)	

*SBP* systolic blood pressure, *DBP* diastolic blood pressure, *MAP* mean arterial pressure, *LDL* low-density lipoprotein, *WBC* white blood cells, PT prothrombin time, *INR* international normalized ratio, *APTT* activated partial thromboplastin time, *NIHSS* national institutes of health stroke scale, *ICH* intracerebral hemorrhage

Based on the multivariate logistic regression analysis presented in Table 3, several factors were found to have a significant impact on mortality in patient with hypertensive intracerebral haemorrhage (HICH), including diabetes mellitus (*p* = 0.005, OR = 5.481), NIHSS score (*p* = 0.003, OR = 3.589), bleeding location in infratentorial (*p* < 0.001, OR = 10.494), and ICH score (*p* = 0.002, OR = 2.757).

Other factors, such as fasting blood glucose (FBG) level (*p* = 0.435, OR = 1.703), hemoglobin level (*p* = 0.874, OR = 1.095), prothrombin time (PT) (*p* = 0.159, OR = 2.060), international normalized ratio (INR) (*p* = 0.358, OR = 2.976), activated partial thromboplastin time (APTT) (*p* = 0.365, OR = 0.604), and bleeding volume (*p* = 0.634, OR = 0.711) were not found to be significantly associated with mortality. Furthermore, management interventions, such as antihypertensive drugs (*p* = 0.092, OR = 2.301) and antilipidemic agents (*p* = 0.120, OR = 0.326), did not show a significant effect on mortality in HICH patients. Given confidence interval results being quite wide, it is acknowledged that the results are not precise. The Variance Inflation Factor (VIF) between NIHSS, ICH score, and bleeding location is 2.968 which mean usually acceptable correlation.

**Table 3:** Multivariate logistic regression analysis of factors influencing mortality

Variables	Standard Error	OR	95% Cl	*P*-Value
Diabetes				
Yes	0.603	5.481	1.680–17.887	0.005
No				
Fasting blood glucose				
Low	0.682	1.703	0.447–6.485	0.435
Normal				
High				
Hemoglobin				
Normal	0.568	1.095	0.360–3.332	0.874
Low				
PT				
Low	0.513	2.060	0.754–5.632	0.159
Normal				
High				
INR				
Low	1.186	2.976	0.291–30.439	0.358
Normal				
High				
APTT				
Low	0.556	0.604	0.203–1.796	0.365
Normal				
High				
Bleeding Volume				
< 30 ml	0.736	0.711	0.168–3.007	0.634
≥ 30 ml				
NIHSS				
No Stroke Symptoms	0.432	3.589	1.538–8.374	0.003
Minor Stroke				
Moderate Stroke				
Moderate to Severe Stroke				
Severe Stroke				
Bleeding Location				
Infratentorial	0.640	10.494	2.993–36.798	< 0.001
Supratentorial				
ICH score				
0	0.382	2.757	1.448–5.247	0.002
1				
2				
3				
4				
5				
6				
Antihypertensive drugs				
Oral	0.495	2. 301	0.872–6.076	0.092
IV				
Antilipidemia agents				
Yes	0.721	0.326	0.079–1.337	0.120
No				

*PT* prothrombin time, *INR* international normalized ratio, *APTT* activated partial thromboplastin time, *NIHSS* national institutes of health stroke scale, *ICH* intracerebral hemorrhage

## Discussion

This study identified association between diabetes mellitus (DM) and mortality in HICH patients (*p* = 0.005, OR = 5.481), consistent with the findings of Arboix et al., who found that DM independently raises the risk of mortality in patients [16]. Diabetes mellitus exacerbates mortality in intracerebral hemorrhage through a series of complex pathophysiological mechanisms. DM induces oxidative stress and activates matrix metalloproteinase-9 (MMP-9), which damages the integrity of the blood-brain barrier (BBB) and leads to more severe cerebral edema. Additionally, DM is often associated with resistant hypertension and diabetic angiopathy, increasing vascular fragility and the risk of vessel rupture. Patients with DM are also prone to immune dysfunction and nosocomial infections and frequently have cardiovascular comorbidities that worsen clinical outcomes [17]. 

The NIHSS score significantly contributes to mortality (*p* = 0.003, OR = 3.589). Higher NIHSS scores are associated with an increased likelihood of patient death. This finding is consistent with the study by Finocchi et al., which reported that the NIHSS score is a valuable prognostic tool for assessing mortality risk in patients with ICH. The study showed a strong correlation between higher NIHSS scores and increased mortality rates in ICH patients [9]. NIHSS predicts mortality by evaluating various neurological aspects, including consciousness, eye movement, sensory function, and motor function in both hemispheres. Due to its comprehensive assessment scope, the NIHSS can detect neurological deterioration potentially caused by hematoma expansion, such as worsening motor deficits, sensory impairment, or cognitive decline. Since hematoma expansion is often associated with patient deterioration and poor clinical outcomes, the NIHSS’s sensitivity to these changes makes it an effective tool for predicting mortality risk in ICH [18]. 

Bleeding location, specifically infratentorial hemorrhage, was identified has an association with mortality in HICH patients (*p* < 0.001, OR = 10.494). This finding is in line with the study by Patel et al., which reported that patients with infratentorial ICH have worse clinical outcomes and greater severity compared to those with hemorrhages in other locations [19]. Hemorrhage in the infratentorial region, such as the brainstem and cerebellum, rapidly increases intracranial pressure due to the limited anatomical space. This elevates the risk of brain herniation, which can disrupt cerebral blood flow, cause ischemia, and compress the brainstem, the area responsible for controlling vital functions such as respiration and heart rate. If left untreated, this condition can result in respiratory failure, cardiac arrest, and ultimately death [20]. 

The ICH score was significantly associated with mortality (*p* = 0.002, OR = 2.757). This finding is consistent with the study by Sitohang et al., which demonstrated that the ICH score is not only significantly associated with 30-day mortality in patients with hemorrhagic stroke, but also has high predictive accuracy for such outcomes [10]. Patients with higher scores indicate greater severity and an increased risk of mortality [21]. The Intracerebral Hemorrhage (ICH) score is a prognostic tool used to predict 30-day mortality in patients with intracerebral hemorrhage. It is based on five key variables: Glasgow Coma Scale (GCS), hematoma volume, age, bleeding location, and intraventricular hemorrhage (IVH) [22]. Low Glasgow Coma Scale (GCS) scores and large hematoma volumes are interrelated factors that increase the risk of mortality, as larger hematomas can cause a significant rise in intracranial pressure, leading to decreased consciousness and a higher risk of brain herniation [22, 23]. Advanced age, particularly ≥ 80 years, worsens prognosis due to reduced physiological brain reserve and comorbidities. Hemorrhage occurring in the infratentorial region, especially in the brainstem and cerebellum, is associated with higher mortality due to the limited anatomical space, where even small bleeds can result in fatal increases in intracranial pressure. Furthermore, the presence of intraventricular hemorrhage (IVH) exacerbates outcomes by increasing the risk of obstructive hydrocephalus and further elevating intracranial pressure [22, 24]. 

In this study, fasting blood glucose (FBG) did not significantly correlate with mortality (*p* = 0.435, OR = 2.703). Previous research has indicated that hyperglycemia independently increases mortality in patients with ICH [16]. Therefore, the findings of this study are not in line with earlier studies, despite FBG being one of the diagnostic parameters for diabetes mellitus (DM), a condition strongly associated with vascular risk, endothelial dysfunction, hematoma expansion, and increased mortality. While fasting blood glucose reflects glycemic status, it may not fully represent the presence or severity of diabetes mellitus. As a transient parameter, fasting blood glucose (FBG) levels are highly influenced by acute conditions such as the stress response to cerebral hemorrhage. Therefore, elevated glucose levels in this context may not indicate chronic diabetes. In other words, FBG may reflect stress-induced hyperglycemia rather than a long-term metabolic disorder associated with atherosclerosis progression that can exacerbate hemorrhage. Thus, to more accurately represent a patient’s diabetic status, parameters that reflect chronic glycemic control, such as glycated hemoglobin (HbA1c), which reflects average glucose levels over the previous 2–3 months, are more appropriate [16, 25]. 

Hemoglobin levels did not significantly affect mortality in this study (*p* = 0.283, OR = 1.824). Previous research, including a meta-analysis, has indicated that anemia at hospital admission is associated with increased mortality within 30 days to 12 months [25]. This discrepancy may be attributed to differences in the study population characteristics, where the number of patients with severe anemia was relatively low, thus diminishing its potential impact on mortality outcomes.

Prothrombin Time (PT), Activated Partial Thromboplastin Time (APTT), and International Normalized Ratio (INR) did not show a significant association with patient mortality in this study, with *p*-values of 0.159 (OR = 2.060) for PT, 0.365 (OR = 0.604) for APTT, and 0.358 (OR = 22.976) for INR, respectively. These findings suggest that these coagulation parameters did not significantly increase risk of mortality. This result contrasts with previous studies, which reported that elevated coagulation parameters such as PT, APTT, and INR are associated with coagulopathy, leading to hematoma expansion, increased risk of rebleeding, and significantly higher mortality [11, 13]. The lack of significance in this study may be attributed to the characteristics of the study population, as patients with severe coagulopathy were excluded, resulting in a narrower variability of PT, APTT, and INR values.

Bleeding volume did not significantly affect mortality (*p* = 0.634, OR = 0.711). This finding is inconsistent with several previous studies indicating that hematoma volume is a strong predictor of 30-day mortality in patients with intracerebral hemorrhage (ICH) [26]. One possible explanation for this discrepancy is that the categorization of bleeding volume in this study may have been too simplistic to capture the clinical complexity of the studied population, despite using internationally recognized cut-off values, such as > 30 cc. Patient-specific factors such as hemorrhage location, age, neurological status upon admission, and differences in medical management may influence the relationship between hematoma volume and mortality. Relying on a single cut-off may overlook the nuanced clinical effects of varying hematoma sizes, thus reducing the sensitivity of the analysis to detect significant associations. A more detailed categorization approach or a continuous variable analysis may provide a more accurate representation of the impact of hematoma volume on mortality in this population. Additionally, the exclusion criteria applied in this study, such as the omission of patients with vascular malformations or blood dyscrasias, likely led to a more homogeneous study cohort, potentially diminishing the relative impact of bleeding volume compared to other prognostic factors.

The use of intravenous antihypertensive agents in this study did not show a statistically significant association with mortality (*p* = 0.092, OR = 2.301). Although there was a tendency toward higher mortality risk among patients receiving intravenous therapy compared to those receiving oral treatment, the result was not statistically robust enough to be considered an independent predictive factor. This finding is not entirely consistent with previous studies that reported nicardipine to reduce mortality risk. However, it is not entirely contradictory either, as those studies also noted that overly aggressive blood pressure reduction through intravenous therapy could lead to hypoperfusion and, consequently, increased mortality [26]. These results suggest that the effect of intravenous antihypertensive therapy on mortality remains unclear and may depend on various factors, including the patient’s clinical condition and the blood pressure management strategy employed.

Although the use of antilipidemic drugs showed a *p*-value of < 0.001 in the bivariate analysis and was considered for multivariate analysis, the final results indicated that the association was not statistically significant (*p* = 0.120). A study by Endres et al. reported that statin use during hospitalization was associated with reduced mortality [14]. However, the findings in this study do not align with those results, as no significant relationship was found between antilipidemic drug use and mortality. This discrepancy may be attributed to this study’s more heterogeneous patient characteristics, where disease severity and other dominant risk factors influenced mortality, thereby obscuring the protective effect of antilipidemic therapy. Additionally, most patients in this study likely initiated antilipidemic treatment during hospitalization, unlike patients on long-term therapy prior to admission. The duration and indication for initial drug administration may impact its effectiveness in reducing mortality risk. These factors may explain why the significant association observed in the bivariate analysis lost significance after adjusting for other variables in the multivariate model.

The results of this study indicate that risk factors for mortality in Hypertensive Intracerebral Hemorrhage (HICH), such as diabetes mellitus, NIHSS score, bleeding location, and ICH score, a line with findings from international findings. However, other factors commonly associated with mortality in previous studies like bleeding volume, hypertension, hyperglycemia, hemoglobin levels, platelet count, prothrombin time (PT), activated partial thromboplastin time (APTT), and international normalized ratio (INR) did not show significant associations in this study. Additionally, external studies suggest that therapeutic interventions, including blood pressure control, lipid-lowering therapy, glucose-lowering medications, and surgical procedures may impact mortality in HICH patients.

### Limitation of study

The discrepancies in results may stem from limitations in this study. Firstly, the retrospective design may hinder causal validity and be prone to data errors. Secondly, the lack of follow-up data hinder assessing long-term outcomes HICH survivors. Thirdly, using data from a single hospital, limits the findings’ generalizability. Fourthly, selection bias and information inaccuracies, especially in patient selection and medical records, could have affected the analysis. Lastly, study’s scope was restricted with angiogram data being absent. Thus absence makes it difficult to confirm if hypertension caused HICH or if it was response to cerebral hemorrhage. Including angiogram data, in future research is crucial to delve deeper in to the relationship between hypertension and HICH. In conclusion, this study sheds light on mortality risk factors in HCIH but underscores the need for further research to explore the complex interplay between hypertension and HCIH.

## Conclusion

The factors significantly influencing mortality were diabetes mellitus, NIHSS score, infratentorial location of bleeding, and ICH score. The findings are expected to assist in the early identification of mortality risk factors in HICH patients, thereby enabling more optimal interventions to enhance clinical outcomes and reduce mortality rates.

## Supplementary Information

Supplementary Material 1.

## Data Availability

All data generated or analysed during this study are included in this published article [supplementary information files research data subject].
