# Supplementary material for: Predictors of mortality of Hypertensive Intracerebral Hemorrhage (HICH) patients: a single centre study
Source: BMC Neurol. 2026 Apr 9;26:333. doi: 10.1186/s12883-026-04847-z (PMC13188812; doi:10.1186/s12883-026-04847-z)
Supplement: Supplementary file 1 — Supplementary Material 1. [file 12883_2026_4847_MOESM1_ESM.pdf]

Hasil	Hidup	0
	Meninggal	1
Usia	25 - 43	0
	44 - 59	1
	60 - 90	2
	> 90	3
JK	Perempuan	0
	Laki-laki	1

SBP	Normal	0
	Pre-HTN	1
	HTN 1	2
	HTN 2	3
DBP	Normal	0
	Pre-HTN	1
	HTN 1	2
	HTN 2	3
MAP	Normal	0
	Pre-HTN	1
	HTN 1	2
	HTN 2	3
Diabetes	Ya	1
	Tidak	0
GDP	Rendah	0
	Normal	1
	Tinggi	2
LDL	Optimal	0
	Diatas normal	1
	Batas tinggi	2
	Tinggi	3
	Sangat tinggi	4
WBC	Normal	0
	Tinggi	1
Trombosit	Normal	0
	Rendah	1
HB	Normal	0
	Rendah	1
PT	Rendah	0
	Normal	1
	Tinggi	2
INR	Rendah	0
	Normal	1
	Tinggi	2
APTT	Rendah	0
	Normal	1
	Tinggi	2
NIHSS	Tanpa stroke	0
	Stroke ringan	1
	Stroke sedang	2
	Stroke sedang/berat	3
	Stroke berat	4

Volume perdarahan	**< 30 ml**	0
	**≥ 30 ml**	1
Lokasi perdarahan	Supratentorial	0
	Infratentorial	1
ICH score	0	0
	1	1
	2	2
	3	3
	4	4
	5	5
	6	6
Antihypertensive drugs	Oral	0
	IV	1
Glucos lowering drugs	Ya	1
	Tidak	0
Antilipidemia agents	Ya	1
	Tidak	0
Operasi	Craniotomy	1
	Vp Shunt	1
	Tidak operasi	0
